# Complete chloroplast genome of *Zanthoxylum avicennae* (Lam.) DC (Rutaceae: *Zanthoxylum*)

**DOI:** 10.1080/23802359.2022.2097894

**Published:** 2022-07-22

**Authors:** Jin-jin Li, Qian Qiu, Fu-qiang Yin, Ming Liu

**Affiliations:** College of Biology and Food Engineering, Chongqing Three Gorges University, Chongqing, China

**Keywords:** *Zanthoxylum avicennae* (Lam.) DC, chloroplast genome, sequence

## Abstract

In this study, the chloroplast (Cp) genome of *Zanthoxylum avicennae* (Lam.) DC was sequenced by high-throughput sequencing technology. The length of the Cp genome of *Zanthoxylum avicennae* was 158,568 bp, and the total GC content was 38.4%, including a large single-copy (LSC) region of 86,318 bp, a small single-copy (SSC) region of 18,250 bp, and 27,000 bp of inverted repeats (IRs). The Cp genome encoded 131 genes, including 88 protein-coding, 37 tRNA, and six rRNA genes. Phylogenetic analysis of the genome sequence showed that *Zanthoxylum avicennae* was closely related to *Zanthoxylum nitidum*, *Zanthoxylum esquirolii* and *Zanthoxylum motuoense* of the Rutaceae family.

*Zanthoxylum avicennae* (Lam.) DC is a deciduous tree species of the Rutaceae family (Zheng et al. 2015), which was first described by Lam in 1861 (Lam 1861). The plants in this genus are accessible as condiments and medicinal agents. It is mainly distributed in the south at about 25° N and grows on flat land, slopes or valleys at low elevations, such as in Taiwan, Fujian, Guangdong, Hainan, Guangxi, Yunnan in China, and other parts of the world such as Africa and northern Vietnam (Schultes 1980). There are a few published studies on *Zanthoxylum avicennae* in China, but most of the studies on this species have focused on the chemical constituents of roots, stems and leaves, and only a few involved genome analysis (Dai et al. 2012).

The leaves, velamen and peel of *Zanthoxylum avicennae* have a strong flavor and are rich in effective chemical components, which dispel wind, remove dampness, activate qi, eliminate phlegm and promote blood circulation to arrest pain (Yu and Jiang 2016). The complete chloroplast (Cp) genome of *Zanthoxylum avicennae* (Lam.) DC remains unknown. Hence, in order to provide a solid foundation for further phylogenetic studies, we assembled and characterized the complete plastome of *Zanthoxylum avicennae* in this study. The present findings will facilitate further investigations of the genome and the phylogenetic relationships of the Rutaceae family.

Fresh and healthy leaves (107°17′90. 97′′E, 31°87′47. 80′′N) were collected from Bazhong in Sichuan Province. In the study, the specimens of *Z. avicennae* were stored in the herbarium of the College of Biology and Food Engineering, Chongqing Three Gorges University (https://www.sanxiau.edu.cn/smkx/, Contact person: Nong ZHOU, Email: erhaizn@126.com), with the voucher number ZQ311516. Total genomic DNA was extracted according to a previously reported modified CTAB method (Yang et al. 2014) and sequenced by second-generation sequencing using Illumina HiSeq 2500 platform (Novogene, Tianjin, China). Trimmomatic v.0.32 software with default parameters is used to filter the original reads to decrease the redundant data. (Bolger et al. 2014). Thereafter, the obtained clean reads were assembled into circular contigs using GetOrganelle (Jin et al. 2020), with *Murraya paniculata* (No. NC_052700) as the reference. Finally, the Cp DNA was annotated by the Dual Organellar GenoMe Annotator GeSeq (Tillich et al. 2017) and CpGAVAS2 (Shi et al. 2019). The annotated Cp genome was submitted to the GenBank (Accession number: OL979477).

The total genome of *Z. avicennae* has a typical tetragonal structure and a length of 158,568 bp. It composes of 86,318 bp large single-copy (LSC) region, 17,986 bp small single-copy (SSC) region and 18,250 bp inverted repeat regions (IRA and IRB). The whole GC content is 38.4%. The Cp genome has consisted of 131 genes, including 88 protein-coding, 37 tRNA, and six rRNA genes.

In order to determine the phylogenetic position of *Z. avicenae*, 29 complete chloroplast genomes sequences were obtained from the NCBI database with *Merrillia* and *Murraya* as an outgroup. The genome sequences were aligned with MAFFT version 7.427 (Katoh and Standley 2013), and then the maximum-likelihood (ML) tree was established by the RAxML (Stamatakis 2014) program, with 1000 bootstrap replicates and the GTRGAMMAI model. The results showed that *Z. avicennae* was closely related to *Zanthoxylum nitidum*, *Zanthoxylum esquirolii* and *Zanthoxylum motuoense* of the Rutaceae family (Figure 1).

**Figure 1.**
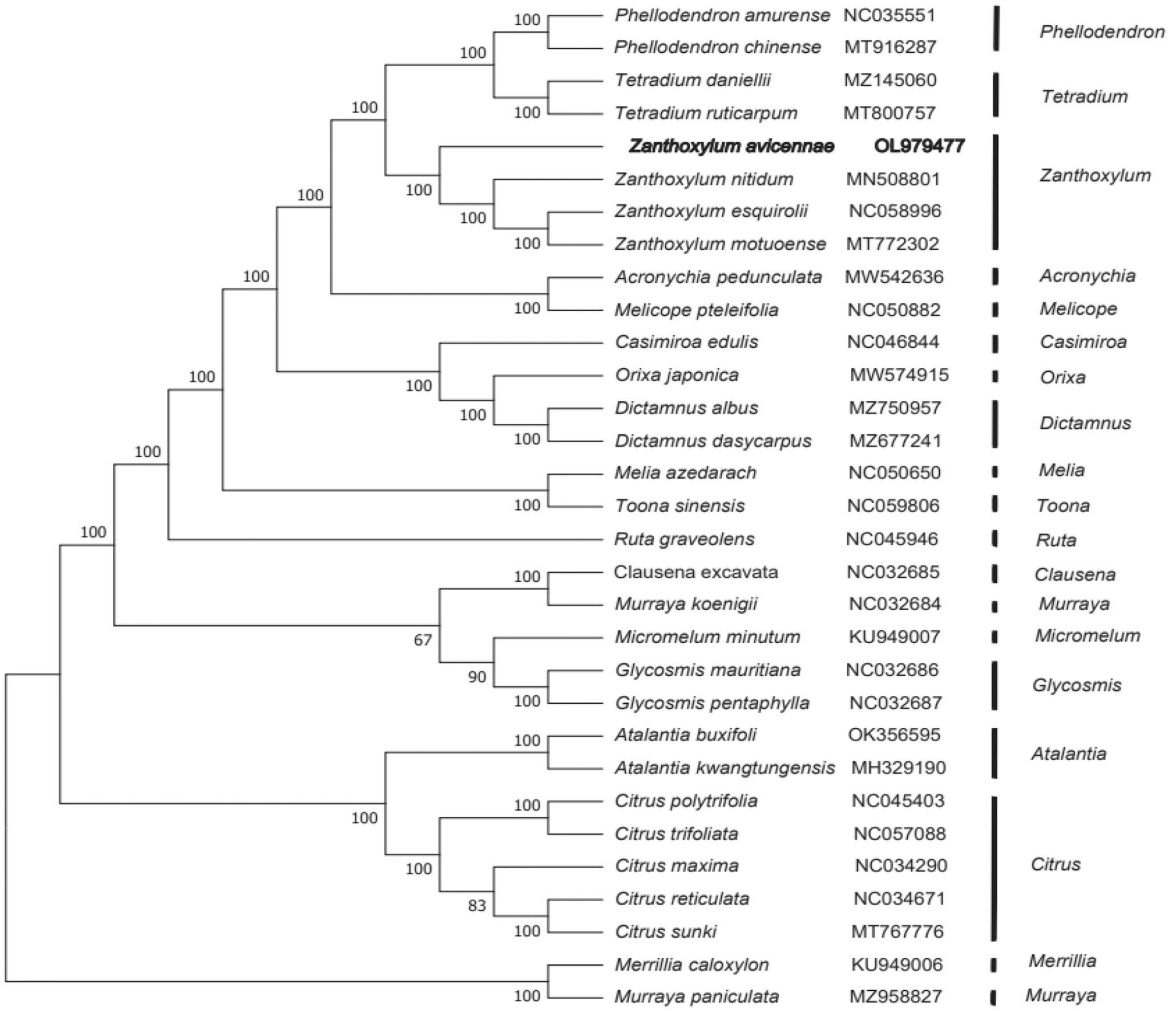
The phylogenetic tree was constructed by maximum-likelihood (ML) analysis based on the complete chloroplast genome sequences of 31 species, with *Ruta graveolens* as an outgroup. *Zanthoxylum avicennae* sequencing was represented by crude linear in this study. Bootstrap support values (1000 replicates) are displayed next to the nodes.

## Data Availability

The data that support the findings of this study are available in GenBank at (https://www.ncbi.nih.gov) under Accession No.OL979477.The associated BioProject, SRA, and BioSample numbersare PRJNA807149, SRR18047727,SRR18047728 and SAMN25965840, respectively.
